# Supplementary material for: Characterization of the First Complete Mitochondrial Genome of Cyphonocerinae (Coleoptera: Lampyridae) with Implications for Phylogeny and Evolution of Fireflies
Source: Insects. 2021 Jun 22;12(7):570. doi: 10.3390/insects12070570 (PMC8307346; doi:10.3390/insects12070570)
Supplement: Supplementary file 1 [file insects-12-00570-s001.zip › insects-1243501-supplementary.pdf]

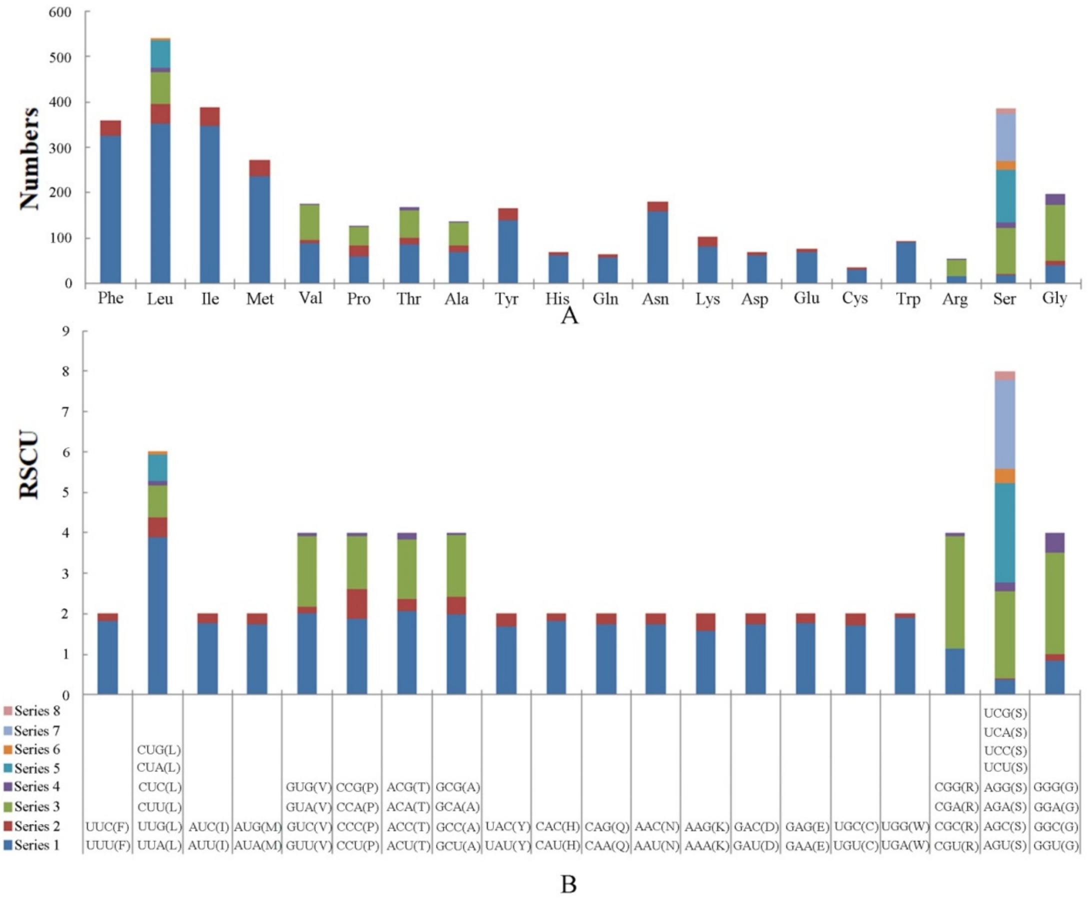

Figure S1 The codon usage numbers (A) and the RSCU (B) of amino acids used for construction of 13 protein coding genes of *C. sanguineus klapperichi*. Codon families are labeled below the X-axis.

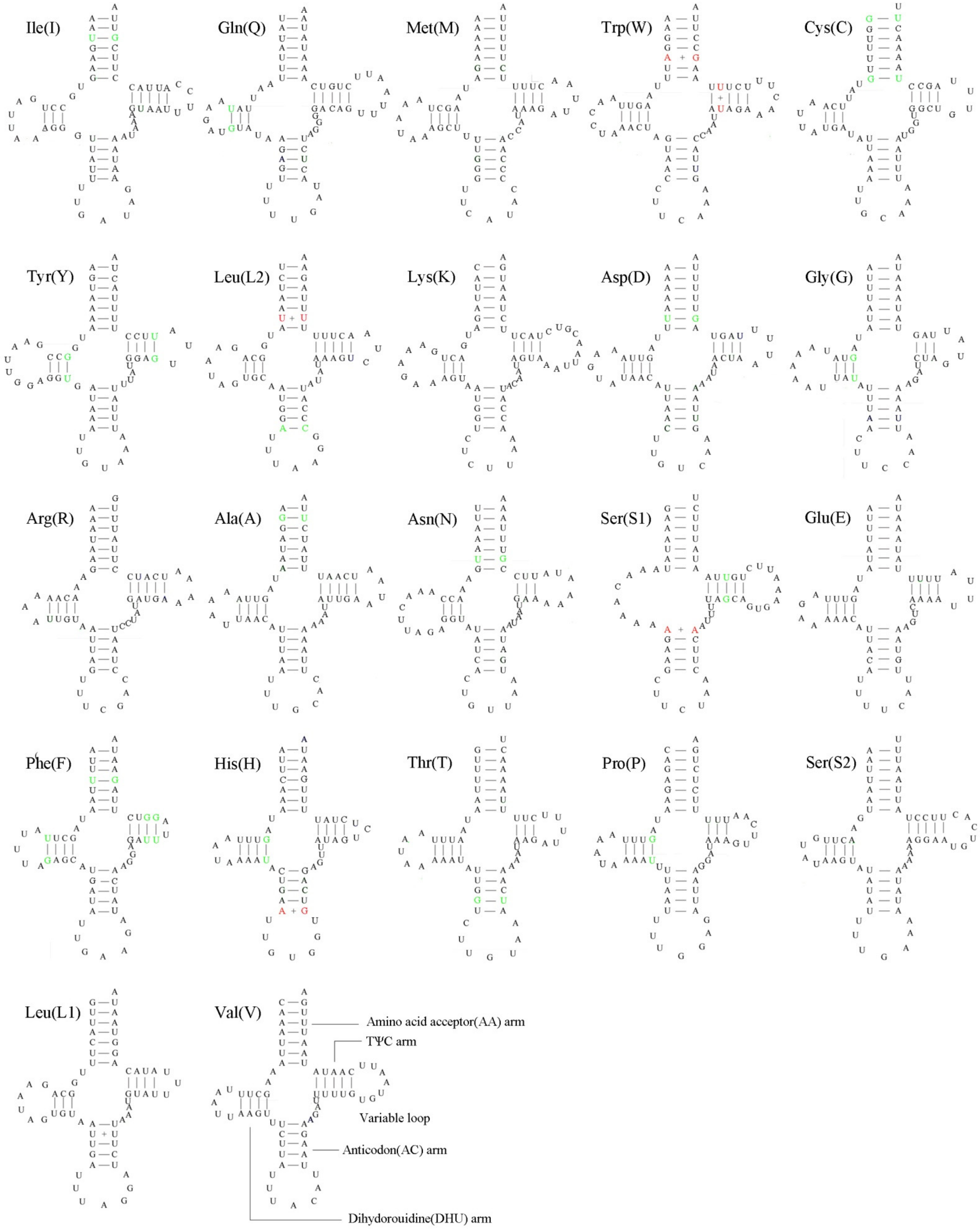

Figure S2 Secondary structures of the tRNAs in the mitochondrial genome of *C. sanguineus klapperichi*. Non-canonical base pairings are indicated in green and mismatched base pairs in red.

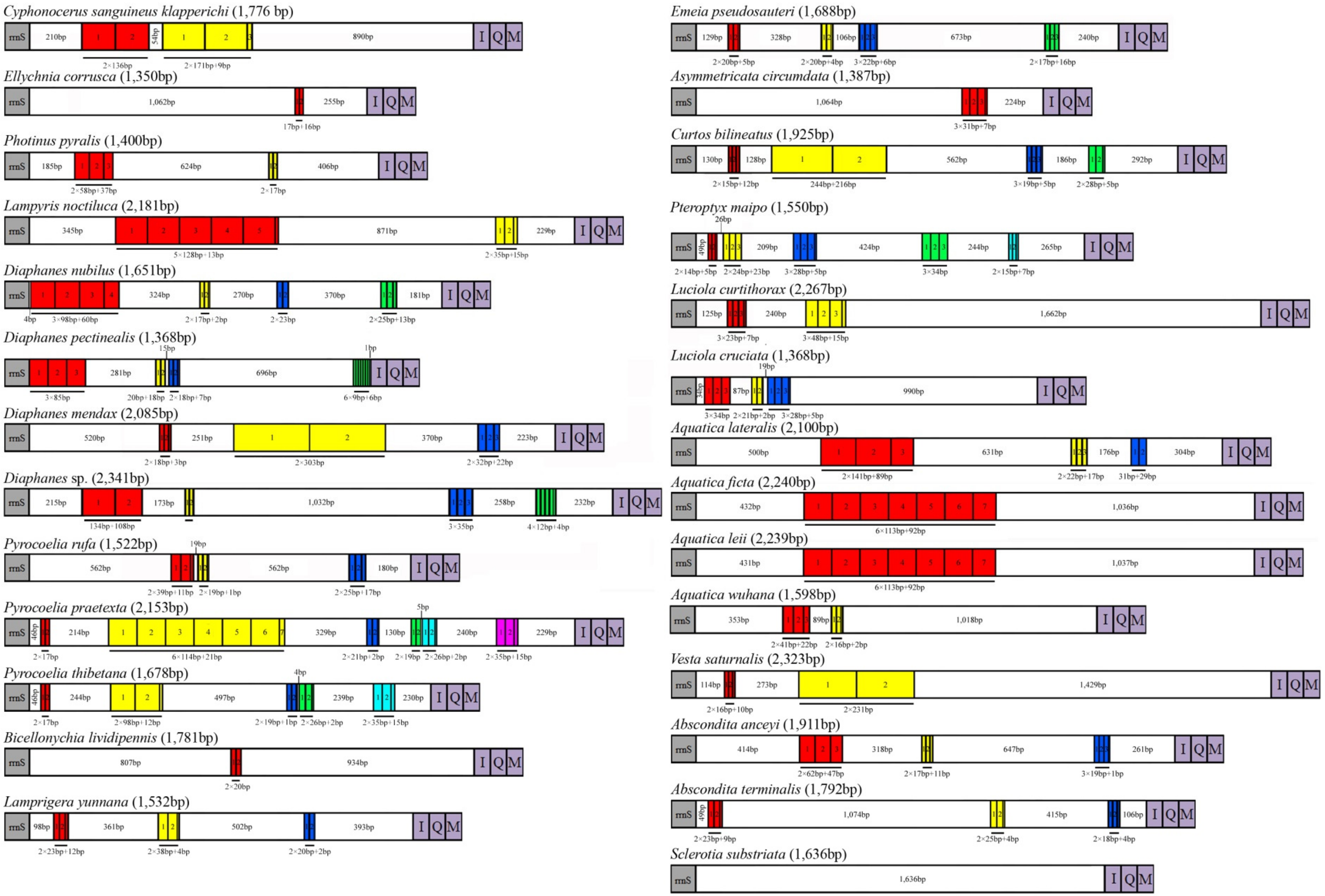

Figure S3 Repeat units of the control region of the mitochondrial genome from *C. sanguineus klapperichi* and the representative species from other firefly subfamilies. Different colors of square bars indicate different tandem repeats, *rrnS* and tRNAs are indicated by the gray and purple square bars, respectively.

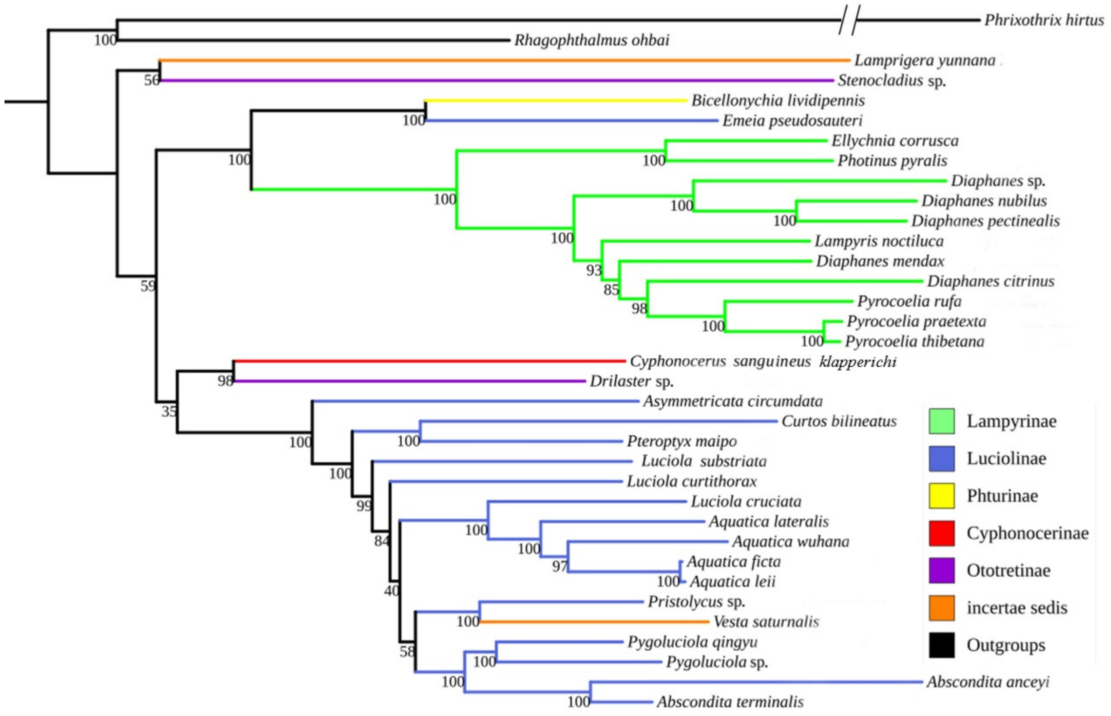

Figure S4 Phylogenetic tree of Lampyridae produced from ML analysis of PCGRNA dataset.

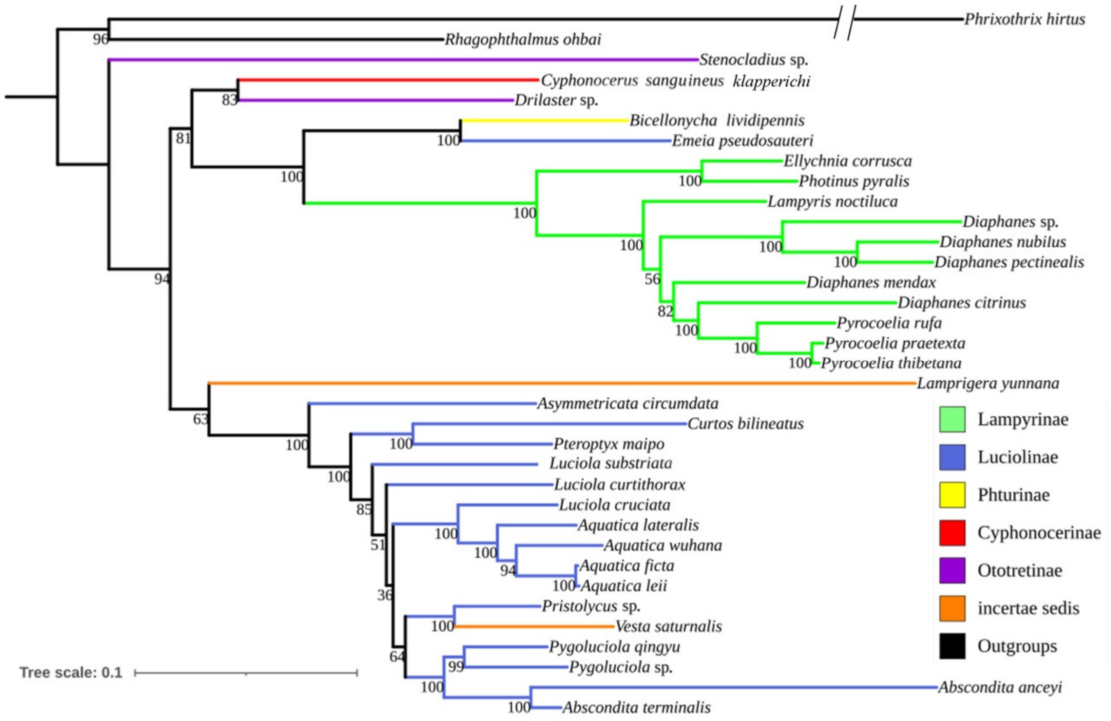

Figure S5 Phylogenetic tree of Lampyridae produced from ML analysis of PCG12rRNA dataset.

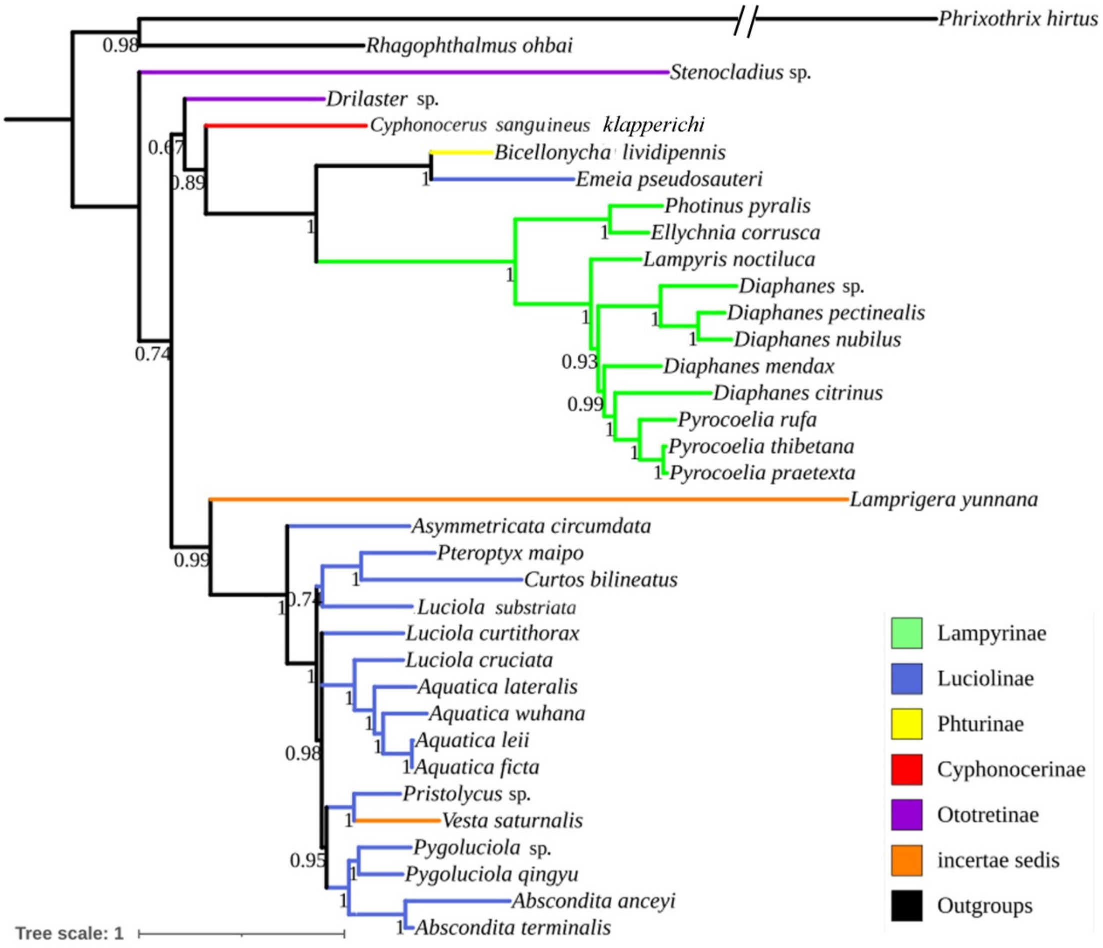

Figure S6 Phylogenetic tree of Lampyridae produced from BI analysis of PCGrRNA dataset.

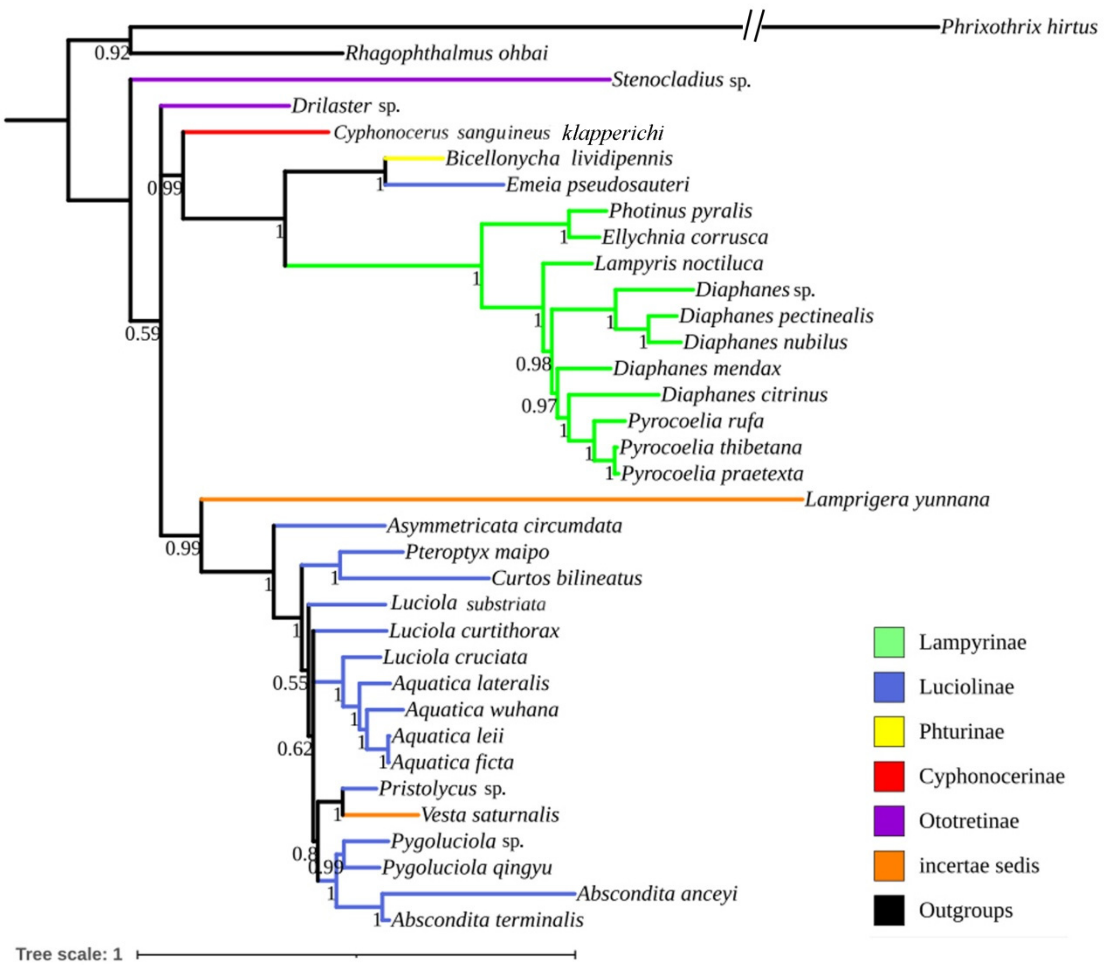

Figure S7 Phylogenetic tree of Lampyridae produced from BI analysis of PCG12rRNA dataset.

Table S1. The best partitioning schemes and models for the Maximum likelihood (ML) method in analysis of the PCG12rRNA matrix

Partitions	Models	Genes
P1	GTR+F+I+G	cox3_pos1, cox2_pos1, cob_pos1, atp6_pos1
P2	GTR+F+I+G	nad6_pos2, nad2_pos2, cob_pos2, nad3_pos2,
P3	GTR+F+I+G	atp8_pos2, atp8_pos1, nad2_pos1, nad3_pos1,
P4	GTR+F+I+G	cox1_pos1
P5	GTR+F+I+G	nad1_pos2, cox1_pos2
P6	GTR+F+I+G	nad4L_pos2, nad5_pos2, cox3_pos2,
P7	GTR+F+I+G	nad5_pos1, nad4L_pos1, nad1_pos1,
P8	GTR+F+I+G	rrnS, rrnL

Table S2. The best partitioning schemes and models for the Maximum likelihood (ML) method in analysis of the PCGrRNA matrix

Partitions	Models	Genes
P1	GTR+F+I+G	cob_pos1, atp6_pos1
P2	GTR+F+I+G	cox2_pos2, atp6_pos2, nad6_pos2, nad2_pos2, nad3_pos2,
P3	GTR+F+I+G	nad3_pos3, atp6_pos3, nad6_pos3, atp8_pos3
P4	GTR+F+I+G	atp8_pos2, atp8_pos1, nad2_pos1, nad6_pos1, nad3_pos1
P5	GTR+F+I+G	cox1_pos1, cox2_pos1, cox3_pos1
P6	GTR+F+I+G	nad1_pos2, cox1_pos2
P7	GTR+F+I+G	cox3_pos3, cob_pos3, cox1_pos3, cox2_pos3
p8	GTR+F+I+G	nad4L_pos2, nad5_pos2, nad4_pos2, cox3_pos2
p9	GTR+F+I+G	nad4L_pos1, nad5_pos1, nad4_pos1, nad1_pos1
p10	GTR+F+I+G	nad4L_pos3, nad1_pos3, nad5_pos3, nad4_pos3
p11	GTR+F+I+G	nad2_pos3
p12	GTR+F+I+G	rrnL, rrnS

Table S3. The best partitioning schemes and models for the Maximum likelihood (ML) method in analysis of the PCGRNA matrix

Partitions	Models	Genes
P1	GTR+F+I+G	rrnL
P2	GTR+F+I+G	rrnS
P3	GTR+F+I+G	tRNA_Leu1
P4	GTR+F+I+G	tRNA_Leu2
P5	GTR+F+I+G	tRNA_Ser1
P6	GTR+F+I+G	tRNA_Ser2
P7	GTR+F+I+G	tRNA_Val
p8	GTR+F+I+G	trnA
p9	GTR+F+I+G	trnC
p10	GTR+F+I+G	trnD
p11	GTR+F+I+G	trnE
p12	GTR+F+I+G	trnF
p13	GTR+F+I+G	trnG
p14	GTR+F+I+G	trnH
p15	GTR+F+I+G	trnI
p16	GTR+F+I+G	trnK
p17	GTR+F+I+G	trnM
p18	GTR+F+I+G	trnN
p19	GTR+F+I+G	trnP
p20	GTR+F+I+G	trnQ
p21	GTR+F+I+G	trnR
p22	GTR+F+I+G	trnT
p23	GTR+F+I+G	trnW
p24	GTR+F+I+G	trnY
p25	GTR+F+I+G	nad6
p26	GTR+F+I+G	nad5
p27	GTR+F+I+G	nad4L
p28	GTR+F+I+G	nad4
p29	GTR+F+I+G	nad3
p30	GTR+F+I+G	nad2
p31	GTR+F+I+G	nad1
p32	GTR+F+I+G	cytb
p33	GTR+F+I+G	cox3
p34	GTR+F+I+G	cox2
p35	GTR+F+I+G	cox1
p36	GTR+F+I+G	atp8
p37	GTR+F+I+G	atp6

Table S4. The best partitioning schemes and models for the Bayesian inference (BI) method in analysis of the PCGRNA matrix

Partitions	Models	Genes
P1	GTR+G	rrnL
P2	GTR+G	rrnS
P3	GTR+G	tRNA Leu1
P4	GTR+I+G	cytb_pos1 ,tRNALeu 2
P5	GTR+G	tRNASer 1, trnR
P6	GTR+I+G	trnD,trnA,tRNASer 2,trnT,trnE,trnG
P7	HKY+I+G	tRNA Val
P8	HKY+I+G	trnQ,trnC
P9	GTR +G	trnI,trnF,trnY
P10	GTR +G	trnP, trnH
P11	GTR +G	trnM, trnK, cox3_pos1
P12	GTR +G	trnN
P13	GTR+I+G	cox2_pos1,trnW
P14	GTR+I+G	nad6_pos1
P15	GTR+I+G	nad6_pos2
P16	HKY +G	nad6_pos3
P17	GTR+I+G	nad5_pos1, nad4_pos1,nad4L_pos1
P18	GTR+I+G	nad5_pos2, nad4_pos2,nad4L_pos2
P19	GTR+I+G	nad5_pos3, nad4_pos3
P20	HKY +G	nad4L_pos3
P21	GTR+I+G	atp8_pos2, nad3_pos1
P22	GTR+I+G	cytb_pos2, nad3_pos2
P23	HKY+I+G	atp8_pos3,atp6_pos3,nad3_pos3
P24	GTR+I+G	nad2_pos1
P25	HKY+I+G	nad2_pos2
P26	GTR +G	nad2_pos3
P27	HKY+I+G	nad1_pos1
P28	GTR+I+G	nad1_pos2
P29	GTR +G	nad1_pos3
P30	HKY+I+G	cox3_pos3,cytb_pos3
P31	GTR+I+G	cox3_pos2
P32	GTR+I+G	cox2_pos2
P33	GTR+I+G	cox1_pos3, cox2_pos3
P34	GTR+I+G	cox1_pos1
P35	GTR+I+G	cox1_pos2
P36	HKY+I+G	atp8_pos1
P37	GTR+I+G	atp6_pos1
P38	GTR+I+G	atp6_pos2

Table S5. Mitogenomic organization of *C. sanguineus klapperichi*

gene	strand	Location	size	Intergenic nucleotides	Stop codon	Start condon	Anticondon
trnI	+	1–66	66				
trnQ	-	64–132	69	-3			
trnM	+	132–197	66				
nad2	+	201–1223	1023	3	TAA	ATA	
trnW	+	1227–1294	68	3			TCA
trnC	-	1367–1429	63	72			GCA
trnY	-	1452–1515	64	22			GTA
cox1	+	1550–3047	1498	34	T	ATT	
trnL2	+	3048–3111	64				TAA
cox2	+	3113–3791	679	1	T	ATG	
trnK	+	3792–3862	71				GTC
trnD	+	3862–3925	64	-1			CTT
atp8	+	3926–4078	153		TAA	ATT	
atp6	+	4075–4746	672	-4	TAA	ATA	
cox3	+	4746–5529	784		TAA	ATG	
trnG	+	5530–5591	62				TCC
nad3	+	5595–5943	349	3	T	ATA	
trnA	+	5944–6008	65				TGC
trnR	+	6009–6073	65				TCG
trnN	+	6074–6140	67				GTT
trnS1	+	6140–6205	66	-1			GCT
trnE	+	6205–6267	63	-1			TTC
trnF	-	6266–6327	62	-2			GAA
nad5	-	6328–8032	1705		T	ATT	
trnH	-	8039–8100	62	6			GTG
nad4	-	8101–9427	1327		T	ATA	
nad4l	-	9424–9681	258	-4	TAA	ATG	
trnT	+	9716–9778	63	34			TGT
trnP	-	9779–9842	64				TGG
nad6	+	9844–10335	492	1	TAA	ATT	
cytb	+	10344–11466	1123	8	T	ATA	
trnS2	+	11467–11533	67				TGA
nad1	-	11551–12495	945	17	TAG	ATT	
trnL1	-	12503–12564	62	7			TAG
rrnL	-	12565–13832	1268				
trnV	-	13833–13901	69				TAC
rrnS	-	13902–14667	766				
CR	+	14668–16443	1776				

Table S6. Comparison of length, nucleotide composition and skewness of the representative fireflies' mitochondrial genomes

Species	Size (bp)	T(U) (%)	C(%)	A(%)	G(%)	AT(%)	GC(%)	A-T(%)	G-C(%)	AT skewness	GC skewness
Abscondita anceyi	16519	36	12	44	8.4	79.8	20.2	7.8	-3.4	0.098	-0.168
Abscondita minalis	16402	35.6	12	44	8.2	79.8	20.2	8.6	-3.8	0.108	-0.188
Aquatica ficta	16836	34.8	14	42	9	77.1	22.8	7.5	-4.8	0.097	-0.211
Aquatica teralis	16719	34.3	14	43	9	76.8	23.2	8.2	-5.2	0.107	-0.224
Aquatica leii	16856	34.8	14	42	9	77.1	22.9	7.5	-4.9	0.097	-0.214
Aquatica wuhana	16186	35.6	13	43	8.6	78.5	21.5	7.3	-4.3	0.093	-0.200
Asymmetricata circumdata	15950	35.4	13	42	8.8	77.8	22.2	7	-4.6	0.090	-0.207
Bicellonycha lividipennis	16466	34.1	15	42	9.6	75.7	24.3	7.5	-5.1	0.099	-0.210
Curtos bilineatus	16677	36.8	12	44	7.6	80.7	19.3	7.1	-4.1	0.088	-0.212
Cyphonocerus sanguineus klapperichi	16443	34.2	14	43	9.4	76.7	23.3	8.3	-4.5	0.108	-0.193
Diaphanes citrinus	>15899	35	12	44	8.7	78.9	21	8.9	-3.6	0.113	-0.171
Diaphanes mendax	17959	34.8	13	43	9.4	77.9	22.1	8.3	-3.3	0.107	-0.149
Diaphanes nubilus	17683	35.7	12	43	10	78.3	21.8	6.9	-1.8	0.088	-0.083
Diaphanes pectinealis	17981	35.6	12	43	9.3	78.5	21.5	7.3	-2.9	0.093	-0.135
Diaphanes sp.	17055	35.4	13	42	9.6	77.5	22.5	6.7	-3.3	0.086	-0.147
Drilaster sp.	>15858	32.3	15	43	9.7	75.1	24.9	11	-5.5	0.140	-0.221
Ellychnia corrusca	16914	35.3	14	40	9.8	75.7	24.2	5.1	-4.6	0.067	-0.190
Emeia pseudosauteri	16327	37	13	42	8.1	79.3	20.8	5.3	-4.6	0.067	-0.221
Lamprigera yunnana	16203	36.2	13	43	7.9	79.3	20.7	6.9	-4.9	0.087	-0.237
Lampyris noctiluca	17221	34.6	14	42	9.4	77	23	7.8	-4.2	0.101	-0.183
Luciola cruciata	15989	32.5	15	43	9.3	75.7	24.2	11	-5.6	0.141	-0.231
Luciola curtithorax	16882	35	12	45	8.2	80	20	10	-3.6	0.125	-0.180
Luciola substriata	16248	34	13	44	9	78.1	21.9	10	-3.9	0.129	-0.178
Photinus pyralis	17081	36.2	14	41	9.4	76.8	23.2	4.4	-4.4	0.057	-0.190

Pristolycus sp.	>14935	34.7	13	43	9.3	77.8	22.1	8.4	-3.5	0.108	-0.158
Pteroptyx maipo	16127	36	12	44	8.2	80.2	19.8	8.2	-3.4	0.102	-0.172
Pygoluciola qingyu	>16413	34.6	12	46	8	80.1	19.8	11	-3.8	0.136	-0.192
Pygoluciola sp.	>15762	34.5	13	43	9.5	77.5	22.5	8.5	-3.5	0.110	-0.156
Pyrocoelia praetexta	17634	34.6	13	42	9.5	77	22.9	7.8	-3.9	0.101	-0.170
Pyrocoelia rufa	17739	34.6	13	43	9.5	77.4	22.6	8.2	-3.6	0.106	-0.159
Pyrocoelia thibetana	18054	34.7	13	42	10	76.4	23.5	7	-3.1	0.092	-0.132
Stenocladius sp.	>15523	32.2	13	46	8.2	78.5	21.6	14	-5.2	0.180	-0.241
Vesta saturnalis	16932	36	12	44	8.3	80.1	19.8	8.1	-3.2	0.101	-0.162

Table S7. Comparison of length and A+T% in different components of mitogenomes of the representative fireflies

Species	PCG		tRNA		*rrnS*		*rrnL*		CR	
	Length	A+T%	Length	A+T%	Length	A+T%	Length	A+T%	Length	A+T%
Abscondita anceyi	11,083	77.7	1,442	80.1	749	81.2	1,267	82.8	1,911	89.5
Abscondita terminalis	11,096	77.4	1,444	79.5	743	81.1	1,265	82.6	1,792	92
Aquatica ficta	11,102	75.3	1,434	77.9	750	79.8	1,263	81.4	2,240	79.6
Aquatica lateralis	11,080	74.1	1,431	78	782	80.8	1,265	81.9	2,100	85.7
Aquatica leii	11,119	75.2	1,436	77.6	750	79.8	1,263	81.4	2,239	82.5
Aquatica wuhana	11,119	76.9	1,426	78.1	746	81.3	1,267	82.2	1,598	85.3
Asymmetricata circumdata	11,084	75.6	1,417	79	742	78.5	1,263	81.8	1,387	89.2
Bicellonycha lividipennis	11,117	73.4	1,436	77.3	772	79.2	1,250	80.3	1,781	83.8
Curtos bilineatus	11,096	78.2	1,425	79.8	742	82.6	1,261	83.1	1,925	92.5
Cyphonocerus sanguineus	11,008	75.1	1,434	77.5	766	78.7	1,268	81.5	1,776	81.2
Diaphanes mendax	11,080	76.1	1,419	79.2	735	81.2	1,245	81.9	2,085	87.3
Diaphanes nubilus	11,073	77	1,414	78.5	728	79.5	1,240	82.4	1,651	86
Diaphanes pectinealis	11,067	76.8	1,413	78	722	79.8	1,242	82.4	1,386	78.1

Diaphanes sp.	11,071	75.3	1,413	78.5	738	80.1	1,241	81.9	2,341	85.6
Ellychnia corrusca	11,054	73.9	1,415	78	738	78.4	1,252	80.2	1,350	86.2
Emeia pseudosauteri	11,116	77.3	1,422	80.2	747	80.6	1,246	83.1	1,688	87.6
Lamprigera yunnana	11,075	77.8	1,399	79.1	741	79.7	1,257	81.8	1,532	87.8
Lampyris noctiluca	11033	74.7	1411	77.6	733	78.5	1241	80.7	2181	87.2
Luciola cruciata	11,016	72.8	1,433	77.4	782	81.6	1,266	81.1	1,368	88.5
Luciola curtithorax	11,085	77	1,469	80.5	743	80.9	1,263	82.9	2,267	86.5
Luciola substriata	11,126	76.3	1,431	78.8	748	79.5	1,268	81.7	1,636	86.2
Photinus pyralis	11,146	75.1	1,424	78.6	736	80.2	1,242	81.3	1,400	87.9
Pteroptyx maipo	11,108	78.3	1,446	80.4	743	81.2	1,264	82.6	1,550	91.3
Pyrocoelia praetexta	11,091	75.3	1,417	78.3	735	81.1	1,245	81.2	2,153	86.2
Pyrocoelia rufa	11,090	76.3	1,412	78.6	765	81.8	1,242	81.6	1,522	87.6
Pyrocoelia thibetana	11,091	75.3	1,415	78.6	734	81.1	1,246	81.3	1,678	88.2
Vesta saturnalis	11,107	78	1,441	78.7	752	82	1,265	82.9	2,323	89.4

Table S8. The count and relative synonymous codon usage (RSCU) of *C. sanguineus klapperichi*

Codon	Count	RSCU	Codon	Count	RSCU	Codon	Count	RSCU	Codon	Count	RSCU
UUU(F)	326	1.81	UCU(S)	117	2.43	UAU(Y)	139	1.68	UGU(C)	30	1.71
UUC(F)	34	0.19	UCC(S)	18	0.37	UAC(Y)	26	0.32	UGC(C)	5	0.29
UUA(L)	352	3.9	UCA(S)	106	2.2	UAA(*)	5	1.67	UGA(W)	90	1.91
UUG(L)	43	0.48	UCG(S)	10	0.21	UAG(*)	1	0.33	UGG(W)	4	0.09
CUU(L)	71	0.79	CCU(P)	60	1.89	CAU(H)	62	1.82	CGU(R)	15	1.15
CUC(L)	9	0.1	CCC(P)	23	0.72	CAC(H)	6	0.18	CGC(R)	0	0
CUA(L)	60	0.66	CCA(P)	42	1.32	CAA(Q)	56	1.75	CGA(R)	36	2.77
CUG(L)	7	0.08	CCG(P)	2	0.06	CAG(Q)	8	0.25	CGG(R)	1	0.08
AUU(I)	346	1.78	ACU(T)	87	2.06	AAU(N)	158	1.75	AGU(S)	18	0.37
AUC(I)	42	0.22	ACC(T)	13	0.31	AAC(N)	23	0.25	AGC(S)	2	0.04
AUA(M)	235	1.73	ACA(T)	62	1.47	AAA(K)	82	1.59	AGA(S)	103	2.14
AUG(M)	37	0.27	ACG(T)	7	0.17	AAG(K)	21	0.41	AGG(S)	11	0.23
GUU(V)	88	2	GCU(A)	68	1.99	GAU(D)	61	1.74	GGU(G)	41	0.84
GUC(V)	8	0.18	GCC(A)	15	0.44	GAC(D)	9	0.26	GGC(G)	8	0.16
GUA(V)	76	1.73	GCA(A)	52	1.52	GAA(E)	68	1.77	GGA(G)	123	2.51
GUG(V)	4	0.09	GCG(A)	2	0.06	GAG(E)	9	0.23	GGG(G)	24	0.49

Table S9. Nucleotide diversity, synonymous and non-synonymous substitution and genetic distance of representative fireflies

Gene	Pi	Ks	Ka	Ka/Ks	Genetic distance
atp6	0.240	0.48983	0.1669	0.3407305	0.291
atp8	0.337	0.41281	0.32056	0.7765316	0.439
cox1	0.187	0.52059	0.08609	0.1653701	0.216
cox2	0.211	0.50315	0.13399	0.2663023	0.254
cox3	0.218	0.49434	0.13943	0.2820528	0.26
cytb	0.225	0.48405	0.15294	0.3159591	0.271
nad1	0.198	0.41508	0.13733	0.3308519	0.234
nad2	0.282	0.4676	0.23408	0.5005988	0.36
nad3	0.257	0.51741	0.18988	0.3669817	0.335
nad4	0.229	0.41075	0.17896	0.4356908	0.278
nad4l	0.221	0.38823	0.17509	0.4509955	0.271
nad5	0.212	0.40722	0.15881	0.3899858	0.265
nad6	0.303	0.42335	0.27128	0.6407937	0.423
